# CLIC1 regulates dendritic cell antigen processing and presentation by modulating phagosome acidification and proteolysis

**DOI:** 10.1242/bio.018119

**Published:** 2016-04-25

**Authors:** Kanin Salao, Lele Jiang, Hui Li, Vicky W.-W. Tsai, Yasmin Husaini, Paul M. G. Curmi, Louise J. Brown, David A. Brown, Samuel N. Breit

**Affiliations:** 1 St Vincent's Centre for Applied Medical Research, St. Vincent's Hospital and University of New South Wales, Sydney, New South Wales 2010, Australia; 2 School of Physics, University of New South Wales, Sydney, New South Wales 2052, Australia; 3 Department of Chemistry and Biomolecular Sciences, Macquarie University, Sydney, New South Wales 2109, Australia

**Keywords:** CLIC1, Dendritic cells, Phagosome, Acidification, Proteolysis, Antigen presentation

## Abstract

Intracellular chloride channel protein 1 (CLIC1) participates in inflammatory processes by regulating macrophage phagosomal functions such as pH and proteolysis. Here, we sought to determine if CLIC1 can regulate adaptive immunity by actions on dendritic cells (DCs), the key professional antigen presenting cells. To do this, we first generated bone marrow-derived DCs (BMDCs) from germline CLIC1 gene-deleted (*CLIC1*^−/−^) and wild-type (*CLIC1*^+/+^) mice, then studied them *in vitro* and *in vivo*. We found phagocytosis triggered cytoplasmic CLIC1 translocation to the phagosomal membrane where it regulated phagosomal pH and proteolysis. Phagosomes from *CLIC1*^−/−^ BMDCs displayed impaired acidification and proteolysis, which could be reproduced if *CLIC1*^+/+^, but not *CLIC1*^−/−^ cells, were treated with IAA94, a CLIC family ion channel blocker. *CLIC1*^−/−^ BMDC displayed reduced *in vitro* antigen processing and presentation of full-length myelin oligodendrocyte glycoprotein (MOG) and reduced MOG-induced experimental autoimmune encephalomyelitis. These data suggest that CLIC1 regulates DC phagosomal pH to ensure optimal processing of antigen for presentation to antigen-specific T-cells. Further, they indicate that CLIC1 is a novel therapeutic target to help reduce the adaptive immune response in autoimmune diseases.

## Abstract (precis)

**Summary:** DC phagosomes from *CLIC1^−/−^* mice display impaired acidification and *in vivo* and *in vitro* antigen processing and presentation, revealing *CLIC1^−/−^* as a potential therapeutic target in reducing the adaptive immune response in autoimmune diseases.

## INTRODUCTION

Antigen presentation is a multiple step processes by which antigen presenting cells (APCs), including macrophages and dendritic cells (DCs), ingest, process and present exogenous antigens, in a complex with MHC class II molecules, to T-cells. APCs first internalize antigen via either endocytosis or phagocytosis, then undertake at least two distinct proteolytic steps. For presentation to CD4 T-cells, there is proteolysis of the antigen and processing of MHC-bound invariant chain (li) to form the class II associated invariant chain peptide (CLIP). If uptake is via phagocytosis, proteolysis of the antigen is initiated by endopeptidases, to fragment the native protein. This is followed by sequential trimming of the peptide ends by amino and carboxypeptidases. This helps to generate small peptides that have the required lengths of 18-20 amino acids (Blum and Cresswell, 1988; Deussing et al., 1998) to sit in the antigen binding groove on MHC class II molecules. Simultaneously, the invariant chain of MHC II undergoes selective proteolytic cleavage of li, which occupies the antigen binding groove. This cleavage event allows for exogenous peptide loading and formation of the MHC II-peptide complex (Busch et al., 2005; Cresswell, 1996), which is then transported to the plasma membrane of APCs.

The major professional APCs are DCs. For DCs to be able to present antigen effectively, following antigen ingestion, they must next undergo maturation (Chow et al., 2002). This can be triggered by activation stimuli from substances including conserved components of microorganisms or pathogen-associated molecular pattern molecules (PAMPs) such as lipopolysaccharide (LPS). PAMPs are recognized by pattern recognition receptors (PRRs) of the innate immune system such as Toll-like receptors (TLRs). Activation of PRR signalling in DCs results in inhibition of further endocytosis or phagocytosis of antigens, upregulation of expression of MHCII molecules and their transport to the cell surface and upregulation of expression of cytokines and costimulatory molecules such as CD40, CD80 and CD86 that are necessary for effective T-cell activation (Trombetta and Mellman, 2005).

Activation and clonal expansion of naïve CD4^+^ T-cells by DCs involves binding of the T-cell receptors (TCR) to MHC II bound antigen on DCs, coupled with binding of the DC expressed costimulatory molecule B7 (CD80/86) to CD28 on T-cells (Huppa and Davis, 2003). In addition, signals provided by distinct cytokines program naïve CD4^+^ T-cells into different T helper (T_H_) subsets including Th1 cells. Activated T_H_1 cells express high levels of intracellular interferon gamma (IFNγ) which is commonly used for flow cytometric detection of T-cell activation, because it is rapidly induced (16 h) after encounter with antigen presented by DCs (Mosmann et al., 1986).

Many studies have demonstrated that proteolysis is an indispensable requirement for effective antigen presentation by DCs (Deussing et al., 1998; Hsing and Rudensky, 2005; Shi et al., 1999). This proteolysis is driven by proteases that reside in the phagosomal or endosomal compartments and function optimally in a defined pH environment. The cathepsins, a class of proteases that are comprised of cysteine and aspartyl proteases, are especially important in processing of peptide for presentation by MHC class II molecules. Most cysteine proteases are unstable and have weak activity at neutral pH and only function optimally in acidic phagosomal compartments. Thus, efficient antigen processing is a highly pH-dependent process.

Unlike macrophages and neutrophils, DCs have developed a more tightly regulated mechanism to sustain their phagosomal pH environment so that peptides are not fully degraded. It is widely accepted that acidification and reactive oxygen species (ROS) production are the two key elements in this regulation. Acidification is mainly, but not exclusively, mediated by the vacuolar ATPase (V-ATPase), which translocate protons from the cytosol into the phagosome lumen (Cross and Segal, 2004). Further, immature DCs have less efficient phagosomal acidification due to limited recruitment of the V-ATPase to lysosomes, as compared to macrophages or mature DCs (Trombetta et al., 2003). Another mechanism that mediates acidification in DCs is the production of ROS from NADPH oxidase 2 (NOX2) leading to an enzymatic multiprotein complex. This multiprotein complex requires the early Rab27a dependent recruitment of gp91*phox* to the phagosomal membrane (Elsen et al., 2004). Rab27a is believed to regulate DC phagosome pH as Rab27a deficient DCs have a delay in the recruitment of NOX2 to the phagosome, resulting in increased phagosomal acidification and antigen degradation, the consequence of which is a defect in antigen presentation (Jancic et al., 2007). Further evidence for the involvement of NOX2 and ROS production in antigen presentation came from work in Vav-deficient DCs. Vav, a member of the guanine nucleotide exchange factor (GEF) family, catalyses the exchange of bound GDP to GTP on Rac, another early component of the NOX2 complex (Crespo et al., 1997). Vav-deficient DCs also showed a decrease in phagosomal pH, an increase in antigen degradation and consequently failed to present antigen efficiently (Jancic et al., 2007; Rybicka et al., 2010). It is believed that the NOX2 complex in DCs produce low levels of ROS, resulting in sustained alkalization of the phagosomal lumen and consequent inefficient antigen processing (Savina et al., 2006).

Recently, we have discovered that intracellular chloride channel protein 1 (CLIC1) regulates macrophage phagosomal pH (Jiang et al., 2012) and thus may also play a role in pH regulation of similar structures in DCs. CLIC1, a member of the evolutionarily conserved 6 member CLIC family of chloride ion channel proteins, was first cloned because of its expression in activated macrophages (Valenzuela et al., 1997). Its gene is located in the MHC class III region of chromosome 6 (Littler et al., 2004) near the gene for TNF-alpha, suggesting a potential role in regulation of immune and inflammatory responses. All protein members of the CLIC family are relatively small in size with only a single putative transmembrane region (Jiang et al., 2014). They are unusual, as they exist in both soluble cytoplasmic and integral membrane forms (Valenzuela et al., 1997). CLIC proteins have to undergo a major structural rearrangement to transform from their glutathione-S transferase (GST)-like structure in the soluble form to that of an integral membrane protein (Goodchild et al., 2009; Littler et al., 2004).

In resting macrophages, CLIC1 resides in uncharacterized cytoplasmic vesicle-like structures. Upon phagocytosis, CLIC1 rapidly translocates to the phagosomal membrane, where it is co-located with other membrane proteins like the Rho GTPases, Rac and RhoA, as well as NADPH oxidase components (Jiang et al., 2012). Using live cell imaging, we have found that *CLIC1*^−/−^ macrophages display impaired phagosome acidification and proteolysis suggesting that CLIC1 may directly regulate phagosomal acidification and as a consequence also proteolysis (Jiang et al., 2012).

Whilst the phagosomal pH of macrophages and DCs are different, they both rely on phagosomal acidification to help regulate proteolysis, which, in DCs, is essential for antigen processing and presentation. In this study we have investigated the role of CLIC1 in regulating these events in bone marrow dendritic cells (BMDCs). We show that in resting BMDCs, CLIC1 is widely distributed in the cytoplasm in punctate vesicle-like structures similar to those we have previously identified in macrophages (Jiang et al., 2012). Shortly after initiation of phagocytosis CLIC1 moves to the BMDC phagosomal membrane where it can be seen with the membrane protein RhoA. Further, we show that *CLIC1*^−/−^ BMDCs have an elevated phagosomal pH which leads to reduced phagosomal proteolysis, the end result of which is reduced *in vivo* and *in vitro* antigen presentation.

## RESULTS

### CLIC1 is present on BMDC phagosomal membranes

**Fig. 1.**
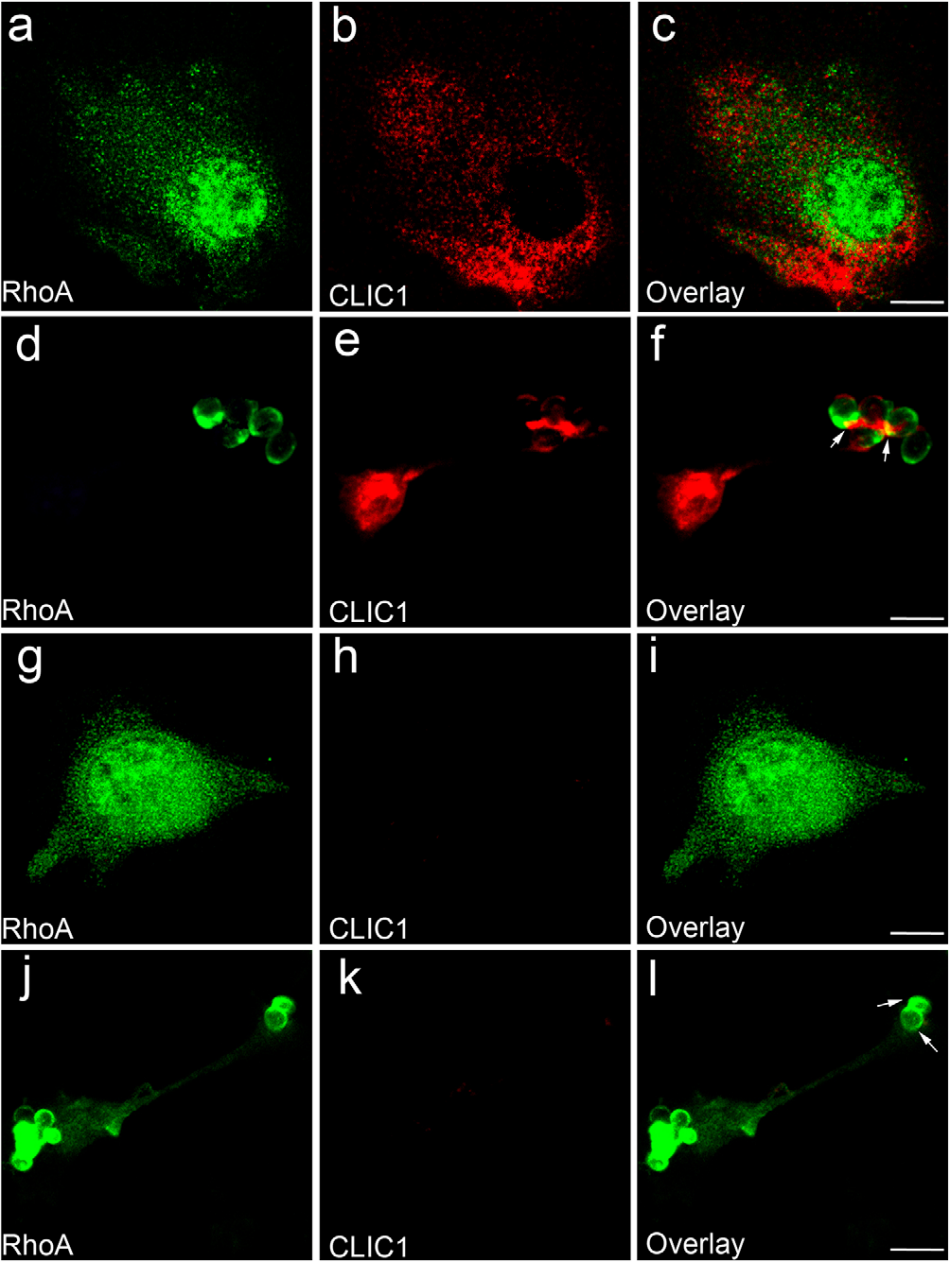
**Phagocytosis triggers CLIC1 translocation to BMDC phagosomal membrane.** (A-F) Immunofluorescence confocal microscopic images of resting *CLIC1*^+/+^ BMDCs (A-C) or BMDCs phagocytosing IgG-opsonised zymosan particles (D-F), stained with antibodies to RhoA (green) or CLIC1 (red). (G-L) Images of resting *CLIC1*^−/−^ BMDCs (G-I) or BMDCs phagocytosing IgG-opsonised zymosan particles (J-L), stained with antibodies to RhoA (green) and CLIC1 (red). Both CLIC1 and RhoA appear on the phagosomal membrane after 5 min of phagocytosis in *CLIC1*^+/+^ BMDCs (F, arrows). Only RhoA can be identified on the phagosomal membrane of *CLIC1*^−/−^ BMDCs (L, arrows). Scale bar: 10 µm.

To determine the subcellular localization of CLIC1 in BMDCs, we have used immunofluorescence confocal microscopy. BMDCs were fixed and stained with an affinity purified sheep polyclonal antibody to murine CLIC1 and a rabbit anti-murine RhoA, followed by a cy3- and cy2-labelled anti-sheep and anti-rabbit IgG, respectively. In resting *CLIC1*^+/+^ BMDCs, CLIC1 staining was punctate (Fig. 1B) in a pattern similar to that we have previously described in macrophages (Jiang et al., 2012). There was a similar staining pattern for the ras homolog family member A (RhoA), which did not co-localise with CLIC1 (Fig. 1A-C). As expected no CLIC1 staining could be identified in *CLIC1*^−/−^ control cells (Fig. 1G-I). To determine whether CLIC1 translocates to phagosomal membranes, 5 min after they had undergone synchronised phagocytosis of IgG opsonized zymosan particles, we fixed then stained BMDCs. RhoA appears on the phagosomal membrane at 5 min (Fig. 1F, arrow), as it is known to do. At the same time point CLIC1 also appears on the phagosome membrane where is partially colocalises with RhoA (Fig. 1D-F). As expected, in *CLIC1*^−/−^ control cells, whilst RhoA staining was present, no CLIC1 antibody staining was detectable (Fig. 1J-L).

### Phagosomes from *CLIC1*^−/−^ BMDCs display impaired acidification

The localization of CLIC1 to phagosomal membranes suggests that it may regulate phagosomal pH in BMDCs. To investigate this, we monitored the process of phagosomal acidification using live cell imaging of *CLIC1*^+/+^ and *CLIC1*^−/−^ BMDCs that had undergone synchronized phagocytosis of IgG opsonised zymosan particles labelled with the pH sensitive dye FITC (zFITC) (Jiang et al., 2012). FITC can effectively differentiate pH values between about 5.5 and 7.5 (Fig. S1) (Chen et al., 2008).

**Fig. 2.**
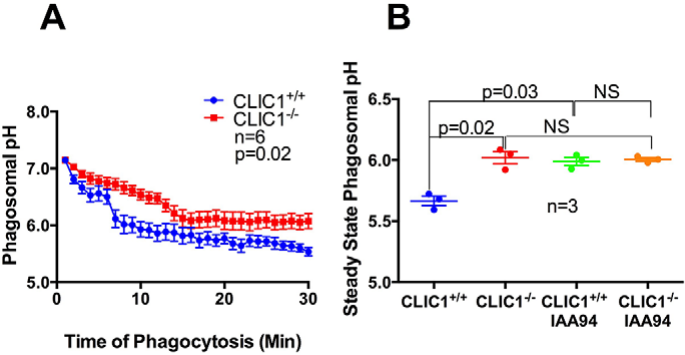
***CLIC1*^−/−^ BMDCs display impaired phagosome acidification.** Live BMDCs that had undergone synchronised phagocytosis of an IgG-opsonised zymosan particle covalently coupled with a pH-sensitive fluorescent probe, in the presence or absence of IAA94 (100 μM), were monitored continuously for 30 min using an inverted Zeiss Axiovert 200 M microscope. (A) The phagosomal pH of *CLIC1*^−/−^ BMDCs was higher than that of *CLIC1*^+/+^ BMDCs over the 30 min time course. (B) IAA94 treatment had no effect on the steady state phagosomal pH of *CLIC1*^−/−^ BMDCs, but impaired the acidification of *CLIC1*^+/+^ BMDC phagosomes to the same level as that of *CLIC1*^−/−^ BMDCs. Data represents mean±s.e.m. analysed using two-way repeated-measures ANOVA or paired *t*-test respectively.

After synchronised phagocytosis, the phagosome of *CLIC1*^+/+^ and *CLIC1*^−/−^ BMDCs slowly acidified (Fig. 2A) with consequent decrease in FITC fluorescence of the phagocytosed particle. The rate of decrease in phagosomal pH of *CLIC1*^+/+^ and *CLIC1*^−/−^ BMDCs started to diverge at about 7 min after phagocytosis. From 7-14 min, the *CLIC1*^−/−^ phagosomal pH clearly dropped more slowly than that of *CLIC1*^+/+^ BMDC phagosomes. Between 15 and 30 min, the phagosomal pH reached a steady state and over this period, the average phagosomal pH of the *CLIC1*^−/−^ cells was higher than that of *CLIC1*^+/+^ cells (Fig. 2B, *n*=6/group with 10-15 zymosan containing phagosomes analysed per experiment; *P*=0.02, two-way repeated-measures ANOVA). These results show that phagosomes from *CLIC1*^−/−^ BMDCs have impaired acidification.

### The CLIC1 ion channel blocker IAA94 raises the pH of *CLIC1*^+/+^ but not *CLIC1*^−/−^ BMDC phagosomes

CLIC1 gene deletion in BMDC leads to impaired phagosome acidification. To help further verify that the impaired acidification was directly due to CLIC1 gene deletion, we treated both *CLIC1*^+/+^ and *CLIC1*^−/−^ BMDCs with IAA94, a small molecule blocker of the CLIC family of ion channels, then monitored phagosomal pH as described above. The average steady state pH of IAA94 treated *CLIC1*^−/−^ BMDCs, calculated based on the average pH between 15-30 min after synchronized phagocytosis, did not differ significantly from untreated *CLIC1*^−/−^ BMDCs [Fig. 2B; pH 6.02±0.05 vs 5.97±0.01 (mean±s.e.m.), *n*=3/group with 10-15 zymosan containing BMDCs analysed per experiment; *P*=0.564, paired *t*-test]. However, IAA94 treatment of *CLIC1*^+/+^ BMDCs significantly raised their average phagosomal pH from 5.63±0.07 to 6.02±0.11 (Fig. 2B; *n*=3/group with 10-15 zymosan containing BMDCs analysed per experiment; *P*=0.03, paired *t*-test). Additionally, the pH of these IAA94 treated *CLIC1*^+/+^ BMDCs was not different from that of CLIC^−/−^ BMDCs (Fig. 2B; pH 5.99±0.03 vs 6.02±0.05, *n*=3/group with 10-15 zymosan containing BMDCs analysed per experiment; *P*=0.648, paired *t*-test). These data indicate that the altered phagosomal pH of *CLIC1*^−/−^ BMDCs is likely to be a direct consequence of gene deletion, and that in our experimental system, the pH effect of IAA94 is due to its specific blockade of CLIC1.

### *CLIC1*^−/−^ BMDC display impaired phagosomal proteolysis

**Fig. 3.**
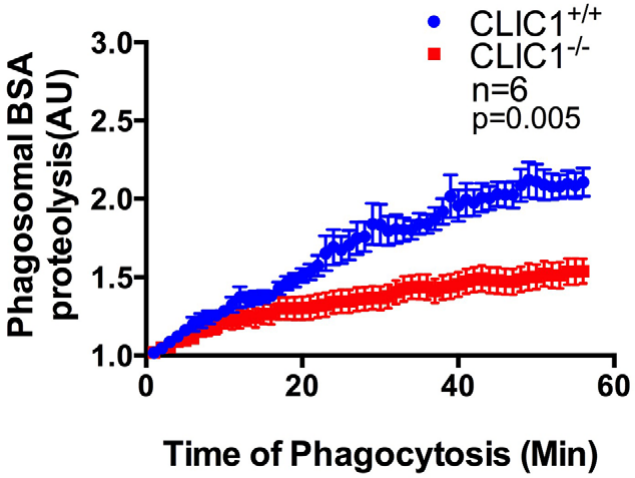
***CLIC1*^−/−^ BMDCs display impaired phagosome proteolysis.** Live BMDCs that had undergone synchronised phagocytosis of 3 µm silica beads, covalently coupled with DQ-bodipy BSA and Alexa Fluor 594, were continuously monitored for 60 min using an inverted Zeiss Axiovert 200M microscope. The time course of proteolytic activity within the phagosome, measured as gain of fluorescence, showed that *CLIC1*^+/+^ BMDCs were more efficient in BSA proteolysis than *CLIC1*^−/−^ BMDCs. Data represents mean±s.e.m. analysed using two-way repeated-measures ANOVA.

Whilst the difference in phagosomal pH between *CLIC1*^+/+^ and *CLIC1*^−/−^ BMDCs is modest, this difference may impact on highly pH-dependent processes such as proteolysis. To directly examine this hypothesis, we used live cell imaging to monitor real time proteolysis in BMDC that had engulfed 3 µm silica beads (Jiang et al., 2012; Yates and Russell, 2008), to which had been coupled Alex Fluor 594 as a reference dye and DQ bodipy BSA as a substrate. The latter becomes more fluorescent as its self-quenching is reduced by proteolysis (Santambrogio et al., 1999). Loosely adhered *CLIC1*^+/+^ and *CLIC1*^−/−^ BMDCs underwent synchronized phagocytosis with the labelled silica beads, which were then monitored by live cell imagining for 60 min. The graph of the change in the fluorescence signal clearly indicates that *CLIC1*^+/+^ BMDCs proteolyse BSA much faster than *CLIC1*^−/−^ BMDCs (Fig. 3; *n*=6/group with 15-20 silica bead containing BMDCs analysed per experiment; *P*=0.005, two-way repeated-measures ANOVA). These rate differences indicate that CLIC1-mediated alteration in phagosomal acidification is also associated with impaired proteolysis in BMDC phagosomes. As proteolysis is a key step in DC antigen presentation, this may have consequences for T-cell activation.

### *CLIC1*^−/−^ BMDC activate fewer T-cells when a large peptide is used as antigen

CLIC1 gene deletion attenuates both DC phagosomal acidification and proteolysis, one consequence of which may be altered DC mediated T-cell activation. To investigate this, we examined the capacity of BMDCs to present myelin oligodendrocyte glycoprotein (MOG) antigen to CD4^+^ T-cells from 2D2 mice which express a transgenic MOG_35-55_ peptide-specific TCR (Bettelli et al., 2003). However, 2D2 mice are on a C57BL/6 background whilst *CLIC1*^−/−^ and control *CLIC1*^+/+^ mice are on a 129X1/SVJ background. Further, as the gene for CLIC1 is in the MHC class III region (Lehner et al., 2004), these mice could not be backcrossed to alter their genetic background to that of C57BL/6. However, C57BL6 and 129X1/SVJ have the same MHCII I-A^b^/I-E^null^ haplotype, which suggests that they may be compatible for our *in vitro* antigen presentation studies. To ensure that, under the conditions of our experiments, no unwanted reactivity was directed by or to 129X1/SVJ cells, we performed a cell mixing experiment. CD4^+^ T-cells were purified from 2D2 mouse spleens using magnetic beads coated with monoclonal antibody to CD4 and were incubated then with BMDCs from 129X1/SVJ or C57BL/6 mice at a ratio of 1:2 BMDC:T-cell for 16 h, with Golgi stop being added for the final 4 h. Activated T-cells, were identified by flow cytometry as CD3^+^CD4^+^CD45^hi^Vβ11^+^ cells (Fig. S2B) that also stained for intracellular INFγ (Fig. S3A). When 129X1/SVJ BMDCs were mixed with 2D2 T-cells, in the absence of antigen, we could identify no difference in T-cell activation from that of C57BL/6 BMDCs (Fig. S3C; 1.43±0.25 vs 1.47±0.34, *n*=3/group; *P*=0.69, unpaired *t*-test). To further confirm there are no unwanted responses, T-cells were also labelled with antibody to CD25/CD69 T-cell activation markers (Fig. S3B). Consistent with INFγ responses, in absence of antigen, there was no evidence of T-cell activation in 129X1/SVJ and C57BL/6 cell mixing experiments (Fig. S3D; 3.77±0.33 vs 4.27±0.20, *n*=3/group; *P*=0.27, unpaired *t*-test). These results exclude any artefact from alloreactivity between 129X1/SVJ BMDC and C57BL/6 2D2 T-cells in our assay format.

To examine the effect of CLIC1 on antigen processing and presentation, *CLIC1*^−/−^ or *CLIC1*^+/+^ BMDCs were to aliquoted into a 96 well plate to which was then added 1.25 pmoles/well of the 21 amino acid MOG peptide MOG_35-55_ or 1.25 pmoles/well of full length recombinant MOG_1-125_ peptide. After incubation of the peptides with BMDCs for various time periods, the cells were washed after which BMDCs were matured and antigen processing stopped by the addition of LPS 0.1 µg/ml for 4 h. MOG-specific 2D2 T-cells (2×10^5^/well) were then added for a further 16 h during which the last 4 h of incubation were in the presence of Golgi stop (1 µg/ml). Activated transgenic T-cells, was then determined by staining for intracellular IFNγ and analysis using multiparameter flow cytometry (Fig. S2B, Fig. S4).

**Fig. 4.**
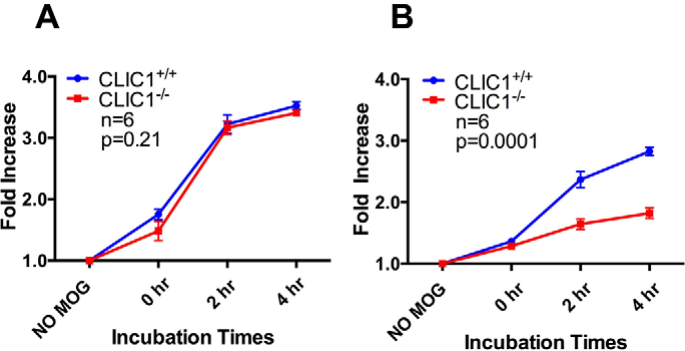
**Antigen**-**pulsed *CLIC1*^−/−^ BMDCs have a reduced capacity to activate CD4^+^ T-cells.** The proportion of activated (intracellular IFNγ-containing) MOG-specific 2D2 CD4^+^ T-cells after 16 h coculture with BMDCs pulsed with (A) 1.25 pmoles of the short MOG_35-55_ peptide or (B) with equimolar full length MOG_1-125_. The fold increase in activated T-cells was calculated relatively to the no antigen control. Data, representing mean±s.e.m., were analysed using two-way repeated-measures ANOVA.

When MOG_35-55_ peptide, that requires no processing to be presented via MHCII was used as an antigen, *CLIC1*^−/−^ and *CLIC1*^+/+^ BMDCs activated similar proportions of T-cells at all time points (Fig. 4A; *n*=6/group with triplicate samples per time point in each experiment; *P*=0.21; two-way repeated-measures ANOVA). However, when BMDCs were pulsed with MOG_1-125_, which does require processing for presentation, *CLIC1*^−/−^ BMDCs activated significantly fewer T- cells at all time points than *CLIC1*^+/+^ BMDCs (Fig. 4B; *n*=6/group with triplicate samples per time point in each experiment; *P*=0.0001, two-way repeated-measures ANOVA). This indicated that if antigen processing is required, *CLIC1*^−/−^ BMDCs present antigen less well than *CLIC1*^+/+^ BMDCs and suggested a potential role for CLIC1 in regulating antigen processing and presentation, which may be mediated, at least in part, by modulation of pH and proteolysis.

### The antigen processing inhibitor chloroquine reduces T-cell activation of a large peptide antigen in *CLIC1*^+/+^ BMDC

Efficient activation of T-cells by large peptide antigens requires both DC antigen processing and presentation to T-cells. To further differentiate these two interdependent processes, we used chloroquine, which inhibits antigen processing by raising the phagosome pH whilst still preserving antigen presentation (Lewinsohn et al., 1998). Using essentially the same experimental procedure as above, *CLIC1*^−/−^ or *CLIC1*^+/+^ BMDCs were preincubated with 100 µl of culture medium containing 100 mM chloroquine. After 1 h, the 21 amino acid MOG_35-55_ peptide or full length recombinant MOG_1-125_ were added to the culture for various time periods after which the cells were matured, 2D2 T-cells were added and the cells stained with the same antibody panel as above, to assess activation of MOG reactive 2D2 T-cells.

**Fig. 5.**
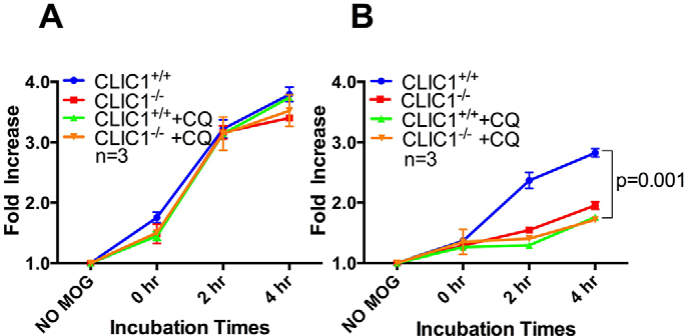
**Chloroquine reduces CD4^+^ T-cell activation by MOG_1-125_**-**pulsed *CLIC1*^+/+^ but not *CLIC1*^−/−^ BMDCs.** The proportion of activated (intracellular IFNγ-containing) MOG-specific 2D2 CD4^+^ T-cells after 16 h coculture with chloroquine (100 μM) treated or untreated BMDCs pulsed with (A) 1.25 pmoles of the short MOG_35-55_ peptide or with (B) equimolar full length MOG_1-125_. Fold increase in activated T-cells was calculated relative to the no antigen control. Data, representing mean±s.e.m., were analysed using two-way repeated-measures ANOVA.

When BMDCs were incubated with MOG_35-55_, which requires little or no processing for efficient antigen presentation, a similar fold increase in activated T-cells were found in *CLIC1*^−/−^ and *CLIC1*^+/+^BMDCs, independent of chloroquine treatment (Fig. 5A; *n*=3/group with triplicate samples per time point in each experiment, *P*=0.15, two-way repeated-measures ANOVA). However, when BMDCs were incubated with full length MOG_1-125_ peptide, which requires antigen processing for effective antigen presentation, the proportion of activated T-cells was very low and similar after incubation with vehicle- or chloroquine-treated *CLIC1*^−/−^ BMDC (Fig. 5B; *n*=3/group with triplicate samples per time point in each experiment, *P*=0.069, two-way repeated-measures ANOVA). Further, antigen presentation by chloroquine-treated *CLIC1*^+/+^ BMDCs was similar to that of the *CLIC1*^−/−^ BMDC but was much lower than that of vehicle-treated *CLIC1*^+/+^ BMDCs (Fig. 5B; *n*=3/group with triplicate samples per time point in each experiment; *P*=0.001, two-way repeated-measures ANOVA). These data further support the hypothesis that CLIC1 is acting to modify antigen processing and therefore reducing the substrate for antigen presentation.

### The CLIC1 ion channel blocker IAA94 diminishes T-cell activation in *CLIC1*^+/+^ but not *CLIC1*^−/−^ BMDCs

To further confirm that CLIC1 gene deletion directly caused the defect in antigen processing, we examined the effect of the CLIC1 chloride ion channel blocker IAA94 (Kim et al., 2004; Pope et al., 1991) on antigen process and presentation. Using essentially the same experimental procedure as above, *CLIC1*^−/−^ or *CLIC1*^+/+^ BMDCs were preincubated in culture medium containing vehicle or 100 mM of IAA94. After 1 h, MOG_35-55_ or MOG_1-125_ was added to the culture for 4 h after which the cells were matured, 2D2 T-cells were then added and 16 h later, and the cells were stained with the same antibody panel as above, for flow cytometric evaluation of 2D2 T-cell activation.

**Fig. 6.**
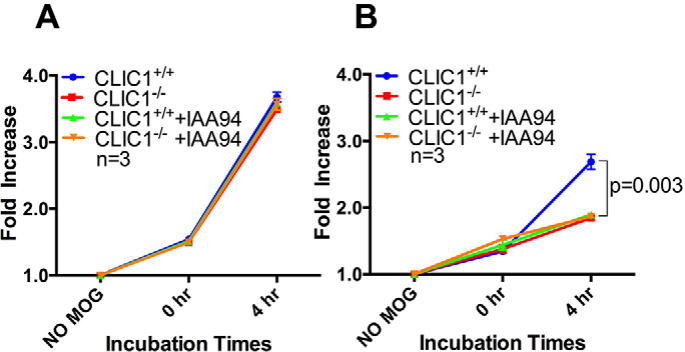
**IAA94 reduces CD4^+^ T-cell activation by MOG_1-125_**-**pulsed *CLIC1*^+/+^ but not *CLIC1*^−/−^ BMDCs.** The proportion of activated (intracellular IFNγ-containing) MOG-specific 2D2 CD4^+^ T-cells after 16 h coculture with IAA94 (100 μM) treated or untreated BMDCs pulsed with (A) 1.25 pmoles of the short MOG_35-55_ peptide or with (B) equimolar full length MOG_1-125_. Fold increase in activated T-cells was calculated relatively to the no antigen control. Data representing mean±s.e.m. were analysed using two-way repeated-measures ANOVA.

Similar to chloroquine, IAA94 did not modify T-cell activation following MOG_35-55_ presentation by either *CLIC1*^+/+^ or *CLIC1*^−/−^ BMDCs (Fig. 6A; *n*=3/group with triplicate samples per time point in each experiment; *P*=0.367, two-way repeated-measures ANOVA). In contrast, when presenting MOG_1-125_, T-cell activation was reduced when *CLIC1*^+/+^ BMDCs were treated with IAA94 (Fig. 6B; *n*=3/group with triplicate samples per time point in each experiment; *P*=0.003, two-way repeated-measures ANOVA). IAA94 treatment of *CLIC1*^+/+^ BMDCs reduced T-cell activation to the same level as vehicle-treated *CLIC1*^−/−^ BMDCs (Fig. 6B; *n*=3/group with triplicate samples per time point in each experiment; *P*=0.282, two-way repeated-measures ANOVA). As expected, IAA94 treatment of MOG_1-125_-pulsed *CLIC1*^−/−^ BMDCs did not alter their capacity to activate T-cells. These data indicate that, by acting specifically on CLIC1, the ion channel blocker IAA94 acts to reduce DC-mediated T-cell activation to MOG_1-125_ which requires processing but not to MOG_35-55_, which requires no processing.

### Reduced T-cell activation by *CLIC1*^−/−^ BMDCs is not due to altered expression of costimulatory molecules

**Fig. 7.**
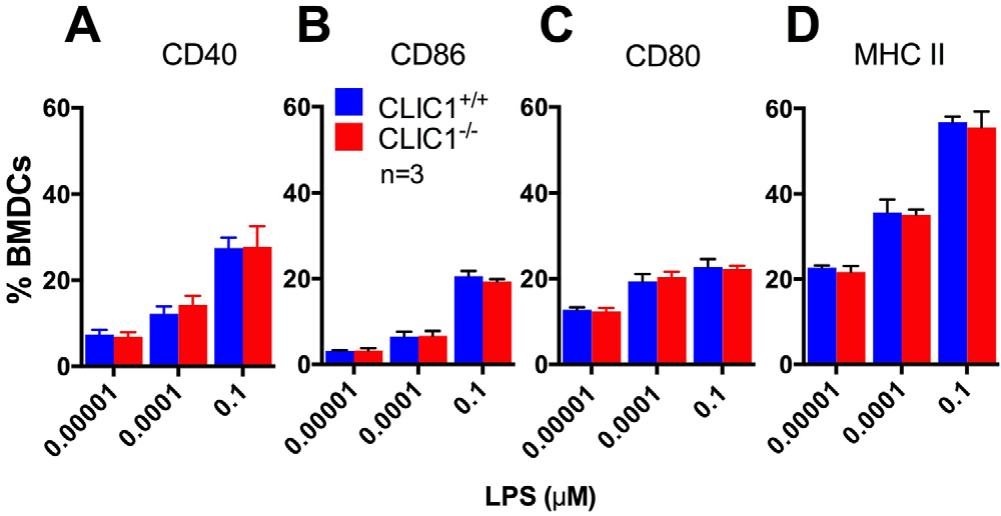
**CLIC1 has no effect on LPS**-**induced BMDC activation cell surface molecules.** The percentage of BMDCs expressing cell surface MHC class II or costimulatory molecules after 4 h incubation with escalating doses of LPS was measured using flow cytometry. There was no significant difference between *CLIC1*^+/+^ and *CLIC1*^−/−^ BMDCs in the proportion of BMDCs expressing CD40 (A), CD86 (B), CD80 (C) or MHC class II molecules (D) for any LPS dose. Data representing mean±s.e.m. were analysed using unpaired *t*-tests.

DC maturation is critical for effective antigen presentation to T-cells, in part because it results in an expression of important costimulatory molecules (De Smedt et al., 1996; Michelsen et al., 2001). To determine if the reduced T-cell activation found on *CLIC1*^−/−^ BMDCs was due to decreases in costimulatory molecule expression, we assessed BMDCs expression of CD40, CD80 and CD86. We also investigated BMDC expression of MHC class II, which is essential for antigen presentation to T-cells. *CLIC1*^−/−^ and *CLIC1*^+/+^ BMDCs (1×10^5^/well) were dispensed into a 96-well plates and then incubated for 4 h with LPS at a concentration of either 0.1, 0.0001 or 0.00001 µg/ml. BMDCs were then washed and stained with antibodies to CD45, CD3, CD11c, CD40, CD80, CD86 and MHC class II. Although expression of these markers was significantly increased with increasing LPS concentration, there was no significant difference in expression of any of these surface markers between *CLIC1*^+/+^ and *CLIC1*^−/−^ BMDCs at any LPS concentration (Fig. 7A-D). This indicates that deletion of CLIC1 gene has no effect on DC expression of CD40, CD80, CD86 and MHC class II and is consistent with the notion that the primary defect is antigen processing, rather than antigen presentation.

### *CLIC1*^−/−^ BMDC presenting MOG_1-125_ induce less EAE disease

Our data strongly indicates that CLIC1 deletion has an effect on BMDC processing of peptide, resulting in a reduced *in vitro* capacity to activate CD4^+^ T-cells. To determine whether these changes could also be demonstrated *in vivo*, we studied murine experimental autoimmune encephalomyelitis (EAE), an established model of multiple sclerosis (Constantinescu et al., 2011). We induced disease in groups of six *CLIC1*^+/+^ and six *CLIC1*^−/−^ age- and sex-matched mice with *CLIC1*^+/+^ or *CLIC1*^−/−^ BMDCs which had been pulsed with MOG_1-125_ and then matured with LPS. The cells were injected subcutaneously (s.c.) into both flanks of *CLIC1*^+/+^ and *CLIC1*^−/−^ mice. One and 3 days later, the animals were also injected with 200 ng of pertussis toxin, which is part of the usual protocol for MOG vaccination-induced EAE (Constantinescu et al., 2011). This microbial product is thought to promote EAE development by facilitating the migration of pathogenic T-cells to the CNS (Hofstetter et al., 2002). The mice were observed daily, in a blinded manner, and disease scores were assigned based on a widely used clinical scoring scale (Constantinescu et al., 2011) ranging from 1 for very mild disease (flaccid tail) to 5 for complete paralysis.

**Fig. 8.**
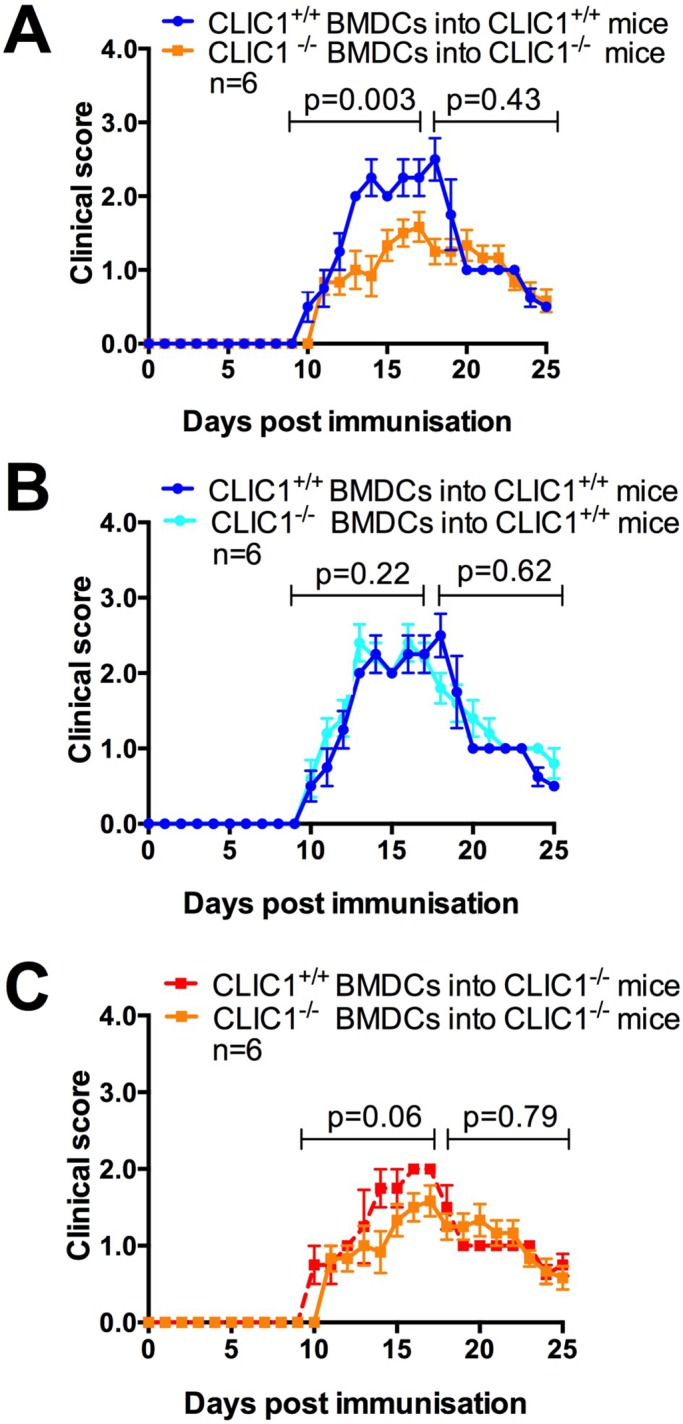
**EAE disease clinical scores of mice immunised with MOG_1-125_**-**pulsed BMDCs.** EAE disease development, measured as clinical scores, was blindly determined in mice immunised with full length MOG_1-125_-pulsed BMDCs. EAE disease development was compared in (A) *CLIC1*^−/−^ and *CLIC1*^+/+^ mice immunised with MOG_1-125_-pulsed *CLIC1*^−/−^ and *CLIC1*^+/+^ BMDC respectively or (B) *CLIC1*^+/+^ mice immunised with MOG_1-125_-pulsed *CLIC1*^−/−^ or *CLIC1*^+/+^ BMDC or (C) *CLIC1*^−/−^ mice immunised with MOG_1-125_-pulsed *CLIC1*^−/−^ or *CLIC1*^+/+^ BMDC. For analysis purposes, the clinical scores have been separated into disease development stage (days 0 to 17) and recovery phase (days 18 to 25) Data, representing mean±s.e.m. scores, were analysed using two-way repeated-measures ANOVA.

From days 9-17, in the disease development phase of EAE, MOG_1-125_-pulsed *CLIC1*^−/−^ BMDCs in *CLIC1*^−/−^ mice elicited less severe EAE than MOG_1-125_-pulsed *CLIC1*^+/+^ BMDCs in *CLIC1*^+/+^ mice (Fig. 8A; *n*=6/group; *P*=0.003, two-way repeated-measures ANOVA). Immunisation of *CLIC1*^+/+^ mice with MOG_1-125_-pulsed *CLIC1*^−/−^ or *CLIC1*^+/+^ BMDCs lead to essentially identical EAE disease severity (Fig. 8B; *n*=6/group; *P*=0.222, two-way repeated-measures ANOVA). However *CLIC1*^−/−^ mice immunised with MOG_1-125_-pulsed *CLIC1*^−/−^ BMDCs compared to *CLIC1*^+/+^ BMDCs, in the disease development phase, displayed less severe EAE disease that fell just short of statistical significance (Fig. 8C; *n*=6/group; *P*=0.064, two-way repeated-measures ANOVA). Overall, these data indicate that *CLIC1*^−/−^ mice have milder EAE disease, and that there is likely to be a reduced capacity of *CLIC1*^−/−^ BMDCs to elicit the initial local response, before secondary amplification of the immune response occurs at more distal sites.

## DISCUSSION

We have found that upon BMDC phagocytosis of an opsonised particle, cytoplasmic CLIC1 rapidly translocates to the phagosomal membrane where it partially colocalises with RhoA, a known phagosome membrane-associated protein. Whilst CLIC1 is on the phagosome membrane, the studies in this manuscript cannot determine if it is an integral or a peripheral membrane protein. However, from this location it regulates phagosomal pH and proteolysis. Whilst CLIC1 modulation of proteolysis is likely to be related to alteration in phagosomal pH, by what means it is altered by CLIC1 is less clear. The most obvious explanation for the increased BMDC phagosomal pH in *CLIC1*^−/−^ mice is lack of CLIC1 chloride ion channel activity on the phagosomal surfaces. Supporting this view is the previous published data demonstrating CLIC protein ion channel activity (Littler et al., 2010b) and in this study, that the CLIC ion channel blocker IAA94 limited phagosome acidification in *CLIC1*^+/+^ BMDCs (Fig. 2B) and impaired their *in vitro* antigen presentation capacity, but had no effect on those functions in *CLIC1*^−/−^ BMDCs (Fig. 6B), indicating that IAA94's actions are specific for CLIC1. Lack of CLIC1-mediated chloride influx would be expected to lead to higher pH values.

Phagocytosis results in progressive phagosomal acidification, an important component of which is V-ATPase proton pump H^+^ influx (Feske et al., 2015). As part of this process chloride ions are needed for charge compensation and many lines of evidence from several groups suggests that CLIC proteins can behave as chloride ion channels (reviewed in Littler et al., 2010b). More recently it has also been shown that mutating key residues in the putative transmembrane domain of CLIC1 markedly modifies its ion channel properties (Averaimo et al., 2013). Further, under oxidizing conditions soluble CLIC1 undergoes a major structural rearrangement (Littler et al., 2004) and in the presence of membranes with cholesterol, monomeric CLIC1 oligomerised to form a pore (Goodchild et al., 2011; Valenzuela et al., 2013). These all suggest that after insertion into a membrane, CLIC1 can form ion channel pores and behave as a chloride ion channel. However, whether CLICs are ion channels is controversial, to a large extent because structural studies of soluble CLICs do not resemble any conventional ion channels but belong to the GST fold superfamily of proteins (Cromer et al., 2007; Harrop et al., 2001; Littler et al., 2005, 2010a) and display glutaredoxin-like glutathione-dependent oxidoreductase enzymatic activity (Al Khamici et al., 2015) and to date, there are no high-resolution structures of the membrane form of CLICs that provide evidence as to how they are able to conduct chloride ions.

CLIC1 might also alter phagosomal pH if it played a role in the phagosome-lysosome fusion. After phagocytosis, phagosomes progressively acidify in parallel with their maturation during which they fuse with other acidic organelles, thereby gaining additional membrane and soluble constituents. The fusion process often requires movement of the phagosomes along microtubules where they can fuse with lysosomes, a process requiring actin assembly at the phagosomal membrane (Blocker et al., 1997). Ezrin, radixin and moesin (ERM) proteins provide a linkage between integral membrane proteins and the actin cytoskeleton (Ivetic and Ridley, 2004) and are downstream effectors of small GTPases (Fehon et al., 2010), which play a part in phagosome–lysosome fusion (Defacque et al., 2000; Erwig et al., 2006; Marion et al., 2011). Whilst there is no direct evidence for CLIC1 in phagosome–lysosome fusion, there are reports that several CLICs interact with ERM. CLIC5 has been purified from placenta microvilli using affinity chromatography with immobilised Ezrin (Berryman and Bretscher, 2000) and in glomerular podocytes, CLIC5A co-localises and can be co-immunoprecipitated with ERM (Pierchala et al., 2010). CLIC4 is also found alongside Ezrin in apical microvilli of retinal pigment epithelium (Chuang et al., 2010). Perhaps the most direct evidences supporting CLIC's role in phagosome-lysosome fusion comes from studies, showing that shortly after macrophage phagocytosis, CLIC3 couples to cytoplasmic domain of a C3b transmembrane receptor (CRIg) on phagosomal membranes which increases chloride conductance into the phagosome lumen, and phagosome–lysosome fusion (Kim et al., 2013). Similarly, in cancer cells, CLIC3 in the late endosome/lysosome compartment works with Rab25 to facilitate recycling of fibronectin binding integrins from late endosome/lysosome to plasma membrane (Dozynkiewicz et al., 2012). Whilst this evidence suggests possible a roles for CLICs in phagosome maturation, using sensitive methods, we have been unable to demonstrate alteration in phagosome–lysosome fusion in *CLIC1*^−/−^ macrophages, that as in BMDCs, also display impaired acidification (Jiang et al., 2012).

Whilst the mechanism of CLIC1 action has not been completely resolved, our studies indicate that phagosomes of *CLIC1*^−/−^ BMDCs, like macrophages (Jiang et al., 2012), display impaired acidification and as a consequence, impaired proteolysis. In DCs, important proteolytic enzymes such as cathepsin proteases and IFNγ-inducible lysosomal thiolreductase (GILT) have actions that are tightly regulated by local pH (Watts, 2012). Consistent with this, impairment of acidification in *CLIC1*^−/−^ BMDC is associated with impairment of antigen presentation of the large MOG_1-125_ peptide, which requires processing, whilst having little effect on the small MOG_35-55_ peptide that does not require processing. This impaired antigen processing could result in reduced adaptive immune responses due to attenuated T-cell responses. To test if our *in vitro* findings would translate into *in vivo* changes we used the MOG-induced EAE model.

To study antigen presentation *in vivo*, we have modified the EAE model by replacing standard immunisation with injection of mice with antigen-pulsed BMDCs. The generation of EAE involves initial antigen presentation in the regional lymph nodes followed by systemic amplification of this immune response in the spleen. Subsequently, there is antigen presentation in the cervical lymph nodes prior to T-cell entry in the CNS (Mohammad et al., 2014). When EAE is induced with *CLIC1*^−/−^ antigen-pulsed BMDCs in CLIC^−/−^ mice there is complete absence of CLIC1 and disease is reduced as expected, compared with the same situation where CLIC1 is replete (Fig. 8A). However, when the same *CLIC1*^−/−^ BMDCs are used to induce EAE in *CLIC1*^+/+^ mice, the situation is more complex. While the initial immune response is likely to be attenuated by *CLIC1*^−/−^ BMDCs, antigen presenting cells in the regional lymph nodes, spleen and cervical lymph nodes have intact CLIC1 and would be expected to present antigen competently, diluting the effect of the initial attenuated immune response. In this pathogenic sequence, it might be expected that normal antigen presentation in the spleen and cervical lymph nodes might lead to equivalent responses in *CLIC1*^+/+^ mice, as we have demonstrated (Fig. 8B). However, when *CLIC1*^+/+^ BMDCs were used to induce disease in *CLIC1*^−/−^ mice, the initial immune response would not be expected to be amplified. Further, differential initial immune responses elicited by *CLIC1*^−/−^ and *CLIC1*^+/+^ BMDCs would be expected to be maintained through the impaired amplification process resulting in differences in disease. Indeed we found that *CLIC1*^−/−^ mice injected with *CLIC1*^−/−^ BMDCs developed less EAE than those injected with *CLIC1*^+/+^ BMDCs, but this just failed to reach significance (Fig. 8C; *P*=0.064). This is likely to be due to a number of factors. The magnitude of disease is less in *CLIC1*^−/−^ mice presumably because of the impaired antigen presentation of key myelomoncytic cell subtypes in these mice, including macrophages and DC, which both contribute significantly to disease (de Vos et al., 2002; Rawji and Yong, 2013). This attenuated disease is likely to significantly reduce the power of our experimental design to detect small differences in disease. Nevertheless, in our experimental paradigm there was an almost significant reduction in EAE between CLIC1-deficient- and replete BMDC-induced disease (Fig. 8C), suggesting the likelihood of reduced *in vivo* antigen presentation by *CLIC1*^−/−^ BMDCs.

Apart from the processing of antigen itself, the impaired phagosomal acidification found in *CLIC1*^−/−^ BMDCs could influence other steps in antigen presentation. Phagosomal proteases are also critical for processing the MHC class II invariant chain (Ii) (Hsing and Rudensky, 2005). However, this is unlikely as Ii processing is required for transport of MHC class II to the cell surface (Fig. 7D) (Watts, 2012) and we found that MHC class II expression was similar on the surface on *CLIC1*^−/−^ and *CLIC1*^+/+^ BMDC cell surface.

Another possible way by which deletion of CLIC1 could result in reduced T-cell activation is by alteration in trafficking of vesicles containing antigen bound MHC class II complexes (MHCII-p), which must translocate to the cell surface for T-cell activation (Roche and Furuta, 2015). However, again, this is unlikely to explain the actions of CLIC1 because *CLIC1*^+/+^ and *CLIC1*^−/−^ BMDCs have similar cell surface staining for MHC class II BMDCs (Fig. 7).

Our results suggest that in DC, CLIC1 regulates phagosomal pH to ensure that the optimal conditions are present for effective antigen processing and presentation and consequent adaptive immune response activation. In the case of autoimmunity, where these processes are dysregulated resulting in immune mediated tissue destruction, CLIC1 may represent a novel therapeutic target.

## MATERIALS AND METHODS

### Chemicals and reagents

The affinity-purified rabbit polyclonal antibody to RhoA is from Abcam (Cat #54853). All secondary antibodies are made in donkey and purchased from Jackson ImmunoResearch Labs. Monoclonal antibodies for flow cytometry were from Beckon Dickinson: anti-CD3-Pacific Blue (clone 1452C11, Cat #558214), anti-CD4-Alex Fluor 700 (clone GK1.5, Cat #557956), anti-CD45-PerCP (clone 30F11, Cat #557235), anti-Vβ11-FITC (clone RR3-15, Cat #553197), anti-CD25-APC-cy7 (clone PC61, Cat #), anti-69-PE-cy7 (clone H1.2F3, Cat #552879), anti-CD86-Alexa Fluor 700 (clone GL1, Cat #560581), anti-CD80-FITC (clone 16-10A1, Cat #553768) and anti-CD40-PE (clone 3/23, Cat #553791). Anti-I-A/I-E-FITC (clone M5/114.15.2, Cat #107606) was from Biolegend and anti-IFNγ-PE-Cy7 (clone XMG1.2, Cat #25-7311-82) was from eBioscience. DAPI was from Invitrogen (Cat #D3571). The short 21 amino acids (MOG_35-55_) and full length 125 amino acid (MOG_1-125_) were from Prospec (Cat #PRO-371) and Anaspec (Cat #55150-1000) respectively. Recombinant murine Flt3-ligand (Flt3, Cat #250-31L), GM-CSF (Cat #315-03) and IL-4 (Cat #214-14) were purchased from Peprotech.

### Mice

All animal work was approved by the Garvan/St Vincent's Hospital animal ethics committee. The germ line gene deleted *CLIC1^−/−^* mice are on a 129X1/SVJ background and have been previously described (Qiu et al., 2010). In all instances, syngeneic 129X1/SVJ mice or cells derived from them were used as *CLIC1*^+/+^ control. 2D2 transgenic mice (C57BL/6 background) were kind gift from Dr Vijay Kuchroo (Harvard Medical School, Boston, MA; Korn et al., 2007).

### Cells and culture medium

All cells were cultured in RPMI-1640 (Cat #11875-093, Life Technologies) containing 100 µg/ml Streptomycin (Cat #15140-122, Life Technologies), 2 mM L-glutamine (Cat #25030-081, Life Technologies), 50 µM 2-Mecaptoethanol, and 10% heat inactivated fetal calf serum FCS (Cat #14190-250, Life Technologies). DCs were generated from bone marrow cells as previously described (Mohammad et al., 2014). MOG specific CD4^+^ T-cells were isolated from lymph nodes from 2D2 mice and purified using magnetic beads as previously described (Mohammad et al., 2014).

### Zymosan preparation

Zymosan (*Saccharomyces cervisiae*) particles (Cat #Z4250, Sigma-Aldrich) were boiled then washed twice in PBS. For opsonisation, 0.5 ml of zymosan particles (14 mg/ml) were mixed with 0.5 ml purified goat IgG (5 μg/ml) (Cat # I9140, Sigma-Aldrich) and incubated at 37°C for 30 min (Jiang et al., 2012). To make FITC conjugated zymosan (zFITC) Zymosan particles were incubated with FITC succinimidyl ester (1 mg/ml, Cat #F-6185, Molecular Probes) (Jiang et al., 2012).

### Bodipy conjugated silica beads

We covalently coupled 3.0 μm carboxylate-modified silica particles (Cat #PSi-3.0COOH, Kisker Products for Biotechnologies) with Alexa Fluor 594 (R) carboxylic acid, succinimidyl ester (mixed isomers) (Alexa594-SE, Cat #A20004, Molecular Probes) and DQ green bodipy bovine serum albumin (DQ-bodipy BSA, Cat #D-12050, Molecular Probes), as described previously (Jiang et al., 2012; Yates and Russell, 2008).

### Immunofluorescence confocal microscopy

Resting BMDCs or BMDCs 5 min after initiation of synchronised phagocytosis were fixed with 4% paraformaldehyde (Cat #C004, ProSciTech) on 8-chamber slides (Cat #354108, BD Biosciences), then permeabilised with 0.05% saponin (Cat #S4521, Sigma-Aldrich) (Jiang et al., 2012). After blocking with 2% IgG free BSA (Cat #001-000-161, Jackson ImmunoResearch Labs) and 1 µg/ml Fc receptor blocking antibody (Cat #553142, BD Biosciences), the cells were stained for; CLIC1 and RhoA. Briefly, BMDCs were firstly stained with 1:100 rabbit anti-mouse RhoA antibody then 1:100 donkey anti-rabbit Cy2 antibody (Cat#ab6940, Abcam) followed by an in-house-derived 1:1000 sheep anti-mouse CLIC1 (Jiang et al., 2012), then 1:100 biotinylated donkey anti-sheep antibody (Cat #713-065-003, Jackson ImmunoResearch Labs) and finally streptavidin Cy3 (Cat#S6402, Sigma-Aldrich) (Jiang et al., 2012). Confocal images were obtained on a Leica TCS SP confocal microscope (Leica Microsystems, Germany) and processed using ImageJ64 (NIH, imagej.nih.gov/ij/download/).

### Intraphagosomal acidification measurement

This was undertaken essentially as previously described (Jiang et al., 2012). Briefly, loosely adherent BMDCs on a fluordish (Cat #FD35-100, Coherent Life Science), underwent synchronized phagocytosis (Jiang et al., 2012) with opsonised zFITCs on the heated stage of microscope stage of a Zeiss Axiovert 200M fluorescence microscope and the particle fluorescence was recorded over 60 min, at a rate of one image per minute (excitation 490 nm, emission 525 nm). In some instances, IAA94 (100 μM) (Cat #I117, Sigma-Aldrich), a cell permeable CLIC1 ion channel blocker, Chloroquine (Cat #C6628, Sigma-Aldrich) or DMSO (Cat #D2650, Sigma-Aldrich) were added to the fluordish. To convert the excitation ratio to pH, time lapse recordings over 45 min were carried out on BMDCs that had phagocytosed opsonised zFITC, incubated in a series of buffers from pH 4 to pH 8 which also contained bafilomycin A1 (100 nM), nigericin (10 µM), valinomycin (10 µM) and carbonyl cyanide m-chlorophenylhydrazone (10 µM) to disrupt membrane channel activity and allow equilibration of intracellular pH with that of the extracellular buffer. There was minimal if any photobleaching (Fig. S1A). A polynomial equation from this data was then derived and used to convert the real time FITC intensity into pH units (Fig. S1B).

### Intraphagosomal proteolysis assay

This was performed essentially as previously described (Jiang et al., 2012). In brief, loosely adherent BMDCs on a 42 mm glass coverslip (Cat #CB00400RA1, Menzel-glaser), underwent synchronized phagocytosis with DQ bodipy-conjugated silica beads on a heated microscope stage, as above. The fluorescence intensities of calibration Alexa Fluor 594 dye (excitation 570 nm, emission 620 nm) and green reporter DQ bodipy dye (excitation 490 nm, emission 525 nm) were acquired over 60 min, at a rate of one image per minute, as described above. The ratio of fluorescence intensity of substrate to calibration fluorescence was plotted against time and used for ratiometric data analysis of intraphagosomal proteolysis.

### Cell mixing experiment and flow cytometry

BMDCs (1×10^5^; 100 μl) from 129X1/SVJ or C57BL/6 mice were mixed with 100 μl of purified CD4^+^ T-cells (2×10^5^) from 2D2 mice in wells of a U bottom 96-well plate (Cat #353077, BD Biosciences) and incubated at 37°C in 5% CO_2_ for 12 h. Golgi stop (1 µg/ml, Cat #554724, BD Biosciences) was added 4 h prior to evaluation of intracellular cytokine staining. Cells were then fixed, permeabilised (Cat #77-5775-40, eBioscience) then stained for with anti-IFNγ-PE-Cy7. In separate experiments, 2D2 CD4^+^ T-cells were also stained for cell surface activation markers using anti-CD25-APC-Cy7 and anit-CD69-PE-Cy7. Flow cytometry data collection was performed on an LSR II (BD Biosciences) and analysed using FlowJo software (Tree Star Inc.).

### *In vitro* T-cell activation

This was undertaken essentially as previously described (Mohammad et al., 2014). Briefly, 1×10^5^ *CLIC1*^+/+^ or *CLIC1*^−/−^ BMDCs in 100 µl of complete medium were incubated with 1.25 pmoles of 21 amino acids MOG_35-55_ or 1.25 pmoles of 125 amino acids MOG_1-125_ peptides or vehicle for up to 4 h at 37°C in 5% CO_2_ in a U bottom 96-well plate, in triplicate. The cells were then washed, and LPS matured after which transgenic 2D2 responder T-cells were added and incubated for a further 16 h. The proportion of activated 2D2 T-cells (positive for CD4, Vβ11 and intracellular INFγ) were identified by flow cytometry using the gating strategy described in Fig. S1B and data were analysed using FlowJo software essentially as previously described (Mohammad et al., 2014).

### Costimulatatory molecule expression

1×10^6^ BMDCs were resuspended into 200 µl complete medium in wells of a U bottom plate containing LPS at 0.1 µg/ml, 0.0001 µg/ml or 0.00001 µg/ml, for 4 h. BMDCs were then washed and stained with 1:100 dilution of anti-CD11c-APC, anti-CD45-PerCP, anti-CD40-PE, anti-CD80-FITC, anti-CD86-Alex700. Cells were also stained with anti-MHC class II-FITC. All antibodies were from BD Biosciences. Appropriate compensation and isotype controls were used. Flow cytometry data collection was performed on an LSR II and analysed using FlowJo software essentially as previously described (Mohammad et al., 2014).

### EAE induction by antigen-pulsed BMDC

Female 8-12-week-old *CLIC1*^+/+^ and *CLIC1*^−/−^ mice were injected subcutaneously in both flanks 50 µl (1×10^6^ cells) with live GM-CSF- and IL-4-generated BMDCs that had been pulsed with 1.25 pmoles of MOG_1-125_.

Myeloid DCs were generated from bone marrow using GM-CSF and IL-4, as described above. BMDCs were then resuspended in 5 ml of complete medium and plated into wells of a 6-well plate (Cat #353046, BD Biosciences) at 1×10^6^ cell/ml. MOG_1-125_ (125 µg/ml; 1.25 pmoles) was added to the cells for 4 h. Cells were washed three times with PBS to remove any residue peptide. LPS (0.1 µg/ml) was added and cells were incubated for another 4 h. These cells were washed and kept on ice at concentration of 1×10^6^ cells in 50 µl. Female 8-12-week-old *CLIC1*^+/+^ and *CLIC1*^−/−^ mice were then injected subcutaneously in both flanks with 50 µl of these BMDCs. Mice were subsequently injected intraperitoneally with 200 ng of pertussia toxin (Cat #180, Sapphire Biosciences) in 0.2 ml PBS at 24 h and 3 days after injection of BMDCs. The mice were then observed daily in a blinded manner, for clinical neurological signs and scores were assigned based on the following widely used scale (Constantinescu et al., 2011) e.g. scale; 1, flaccid tail; 2, hind limb weakness; 3, complete hind limb paralysis; 4, complete hind limb paralysis and forelimb paralysis; 5, complete paralysis.

### Statistical analysis

All data were expressed as mean±s.e.m. Statistical comparisons were performed using Student's *t*-test or two-way repeated-measures ANOVA and post-tested with Bonferroni. *P*-values <0.05 were considered to be statistically significant. All data were analysed using GraphPad Prism 6.0 statistical software.
